# Distinguishing externally from saccade-induced motion in visual cortex

**DOI:** 10.1038/s41586-022-05196-w

**Published:** 2022-09-14

**Authors:** Satoru K. Miura, Massimo Scanziani

**Affiliations:** 1 Center for Neural Circuits and Behavior, Neurobiology Section and Department of Neuroscience, University of California, San Diego, La Jolla, CA USA; 2 Department of Physiology, University of California, San Francisco, CA USA; 3 Howard Hughes Medical Institute, University of California, San Francisco, CA USA

**Keywords:** Neuroscience, Physiology

## Abstract

Distinguishing sensory stimuli caused by changes in the environment from those caused by an animal’s own actions is a hallmark of sensory processing^1^. Saccades are rapid eye movements that shift the image on the retina. How visual systems differentiate motion of the image induced by saccades from actual motion in the environment is not fully understood^2^. Here we discovered that in mouse primary visual cortex (V1) the two types of motion evoke distinct activity patterns. This is because, during saccades, V1 combines the visual input with a strong non-visual input arriving from the thalamic pulvinar nucleus. The non-visual input triggers responses that are specific to the direction of the saccade and the visual input triggers responses that are specific to the direction of the shift of the stimulus on the retina, yet the preferred directions of these two responses are uncorrelated. Thus, the pulvinar input ensures differential V1 responses to external and self-generated motion. Integration of external sensory information with information about body movement may be a general mechanism for sensory cortices to distinguish between self-generated and external stimuli.

## Abstract (web summary)

Distinct activity patterns in the primary visual cortex distinguish movement in the environment from motion caused by eye movements.

## Main

Sensory stimuli are often generated by an animal’s own movements, and nervous systems have evolved mechanisms to distinguish these self-generated stimuli from externally generated ones^1^. Prime examples are saccades, rapid eye movements that induce fast displacement of the visual scene on the retina. They are common in animals across phyla, including in animals without fovea such as rodents, and they contribute to shifts of the gaze^3–6^. Behavioural studies have indicated that such saccade-induced motion of the visual scene is distinguished by subjects from motion occurring in the environment^7–12^.

How visual systems distinguish between the two types of motion, despite similar shifts of the image on the retina, has been a long-standing question. A non-visual, extra-retinal signal occurring around the time of saccades has been proposed to have a key role. This non-visual signal is believed to be transmitted to specific nodes along the visual pathway, where it interacts with the neural responses to saccade-induced motion of the visual scene^13–17^. In visual cortex, it has been proposed that the non-visual signal alters the pattern of the responses to motion^17^, such that the representation of saccade-induced motion of the visual scene is distinct from that of actual motion in the environment. However, the origin of the non-visual signal to the visual cortex, what it encodes and how it impacts the neural representation of the motion induced by saccades is not known.

## Saccade direction preference in V1

**Fig. 1:**
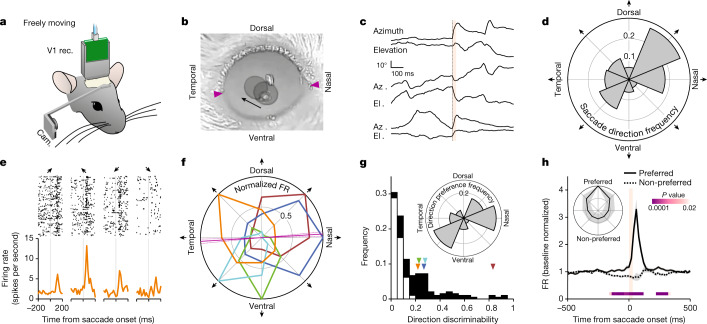
V1 neurons are tuned to saccade direction. **a**, Experimental set-up for eye monitoring in freely moving mice. **b**, Overlay of two snapshots, taken before and after a dorso-temporal saccade (right eye). The arrow indicates the direction of the saccade. The pupils are overlaid with grey circles. Arrowheads indicate temporal and nasal commissures. **c**, Eye position traces for three example saccades. Orange bar, 0–90% rise time of saccades (31 ms). In each pair, the top trace shows azimuth (up is nasal) and the bottom trace shows elevation (up is dorsal). **d**, Polar histogram showing saccade direction frequency. Average of five animals, normalized. **e**, Example V1 neuron showing preference for the dorso-temporal saccade direction. Raster plots (top) and PETHs (bottom) are shown. Arrows indicate the direction of saccades as defined in **f**. **f**, Polar plot of five example saccade direction-selective V1 neurons, normalized to their maximum response (average activity in the 100-ms window after saccade onset). Orange, example neuron in **e**. Magenta line, angle of the axis connecting the temporal and nasal commissures (solid line, average; dotted lines, s.d.). **g**, Direction discriminability of saccade-responsive neurons (based on the receiver operating characteristic of the spike frequency distribution; 0, no discriminability; 1, perfect discriminability;  Methods). Black, direction-selective neurons (*n* = 90 neurons); white, non-selective neurons (*n* = 104 neurons; 10 mice). Arrowheads indicate the discriminability of the example neurons in **f**. Inset, polar histogram of preferred direction frequency. **h**, Average PETH of saccade direction-selective neurons (*n* = 90 neurons, 10 mice) for preferred and non-preferred (that is, opposite) directions. Shaded area, average ± s.e.m. Orange bar, 0–90% rise time of saccades (31 ms). *P* values are from comparison of activity for preferred and non-preferred directions in 20-ms bins (Wilcoxon signed-rank test, one tailed; Methods). Inset, polar plot of the average response of direction-selective neurons, aligned to the preferred direction. Shaded area, s.d.

**Extended Data Fig. 1:**
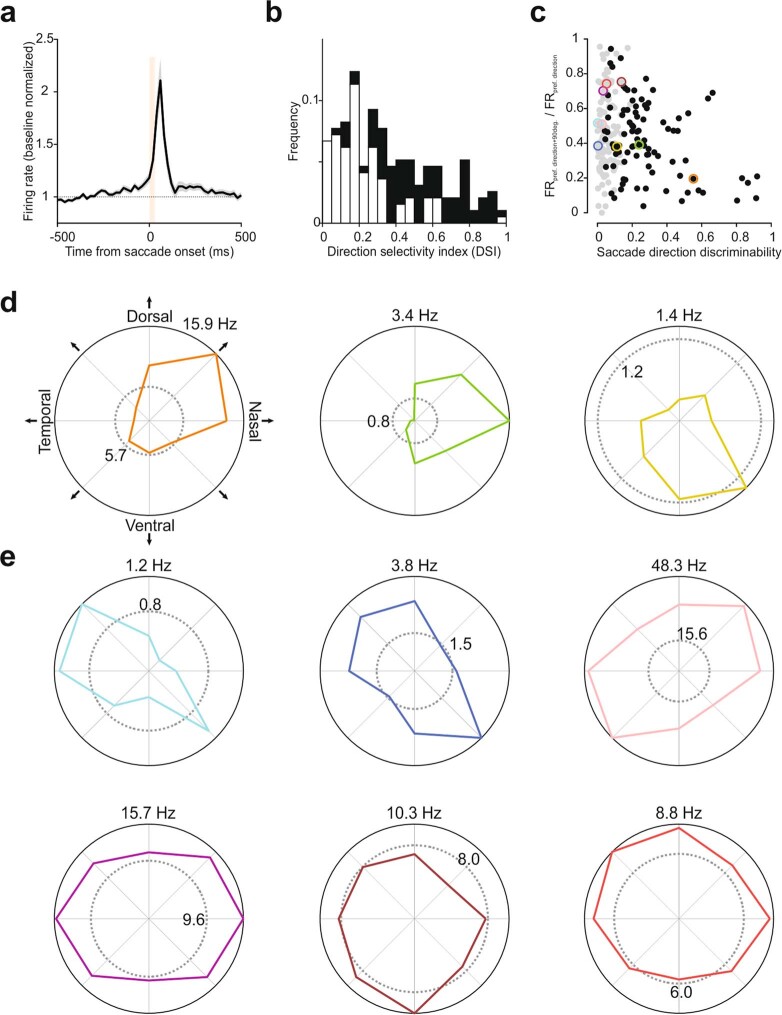
Response of V1 neurons to saccades in freely moving mice. a, Average PETH of saccade responsive neurons (*n* = 194 neurons, 10 mice). For each neuron, the saccade direction with maximum response was taken. Baseline normalized. Shaded area, average ± s.e.m. Vertical orange bar, 0 – 90% rise time of saccades (31 ms). b, Histogram of classical direction selectivity index (see Methods). White, non-direction selective (*n* = 104 neurons); black, saccade direction selective (*n* = 90 neurons). c. Scatter plot of saccade responsive neurons, showing the gradient of response profiles. X-axis, saccade direction discriminability; y-axis, ratio of the FR between the direction *orthogonal* to the preferred and the preferred. Gray, non-direction selective; black, direction selective. Data points in colored circles correspond to example units shown in (d) and (e). d, Polar plots of example saccade direction selective neurons. Dotted gray circle, baseline FR averaged for all directions. FR for the preferred direction is shown at the top of the polar plots. e, Same as (d), but for non-direction selective neurons.

We recorded the response of primary visual cortex (V1) neurons to saccades in unrestrained mice with chronically implanted extracellular electrodes, freely moving in a small illuminated arena. The movements of the eye contralateral to the recorded hemisphere were tracked with a head-mounted miniature camera (Fig. 1a–c). Saccades occurred in all directions yet were biased along the horizontal as compared with the vertical axis (21,981 horizontal and 12,550 vertical saccades from 10 animals; binomial test, *P* < 0.0001; Fig 1d). They had a frequency of 44.2 ± 7.9 events per minute, mean amplitude of 19.0° ± 1.6° and mean 10–90% rise time of 26.8 ± 1.5 ms, resulting in an average speed of 703° ± 49° per second (average ± s.d.) of 10 mice). A large fraction of V1 neurons showed time-locked responses to saccades (194 of 359, 10 mice; Fig. 1e, Extended Data Fig. 1 and Methods), and their responses to saccades both preceded and outlasted saccades by several tens of milliseconds, as shown by the peri-event time histogram (PETH; Fig. 1h). Notably, the response of V1 neurons depended on the direction of the saccade (Fig. 1e–h and Extended Data Fig. 1). While some neurons showed stronger responses to nasal saccades, others preferred ventral, dorsal or temporal saccades or ones of intermediate direction. This directional bias was captured by the discriminability index, a measure of how well an ideal observer can distinguish between the preferred and non-preferred directions of saccades on the basis of spike counts (0, chance level; 1, perfect discrimination; Methods). On the basis of this metric, about half of saccade-responsive neurons discriminated saccade direction (90 of 194; Fig. 1e–h, Extended Data Fig. 1 and Methods). Similarly, about half of saccade-responsive neurons had a direction selectivity index (a classical directional bias metric; Methods) equal to or greater than 0.3 (99 of 194; Extended Data Fig. 1). All saccade directions were represented, yet these were unevenly distributed, with a larger fraction of neurons preferring saccades along the naso-temporal (NT) axis (Rao’s spacing test, *P* < 0.001; Fig. 1g, inset). Taking these findings together, neurons in V1 respond to saccades in a direction-selective manner.

## Non-visual response to saccades in V1

**Fig. 2:**
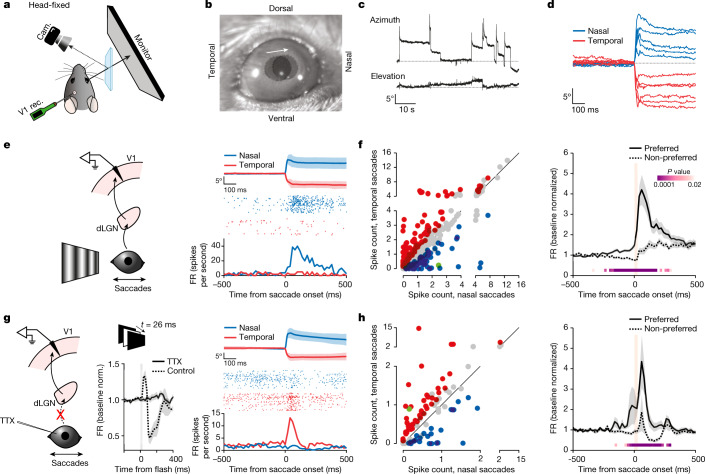
Direction-selective non-visual V1 response to saccades. **a**, Experimental set-up in head-fixed mice. **b**, Two overlaid snapshots (before and after a nasal saccade). The arrow indicates saccade direction. Circles delineate the pupils. **c**, Example eye position traces. Top, azimuth (up is nasal); bottom, elevation (up is dorsal). **d**, Example azimuthal eye position for nasal and temporal saccades. **e**, Left, schematic of V1 recording during saccades on a vertical grating. Right, example neuron. Average eye position for nasal and temporal saccades (top; shaded area, average ± s.d.), raster plots (centre) and the PETH (bottom) are shown. **f**, Left, scatterplot of the response to nasal and temporal saccades (average spike count in a 100-ms window from saccade onset), for all responsive neurons (*n* = 415 neurons, 13 mice). Blue, nasal preference; red, temporal preference; grey, no statistical difference; green, example in **e**. Right, average PETH of discriminating neurons (*n* = 192 neurons, 13 mice), for preferred and non-preferred directions. Shaded area, average ± s.e.m. Orange bar, 0–90% rise time of saccades (26 ms). *P* values are from the comparison of activity for preferred and non-preferred saccade directions in 20-ms bins (Wilcoxon signed-rank test, one tailed). **g**, Left, schematic of V1 recording during saccades in TTX-blinded animals. Centre, average multi-unit responses to a brief full-field flash. Grey bar, flash duration (26 ms). Note the lack of response with TTX. Control, 137.9 ± 31.2 Hz (FR ± s.e.m. averaged over a 60-ms window 10 ms after response onset; *n* = 4 mice; 22.1% ± 4.3% increase over baseline); TTX, −8.0 ± 15.4 Hz (0.12% ± 3.0% increase over baseline; *n* = 8 mice). Wilcoxon rank-sum test, one tailed: *P* = 0.0020. Right, example neuron in a TTX-blinded animal. Average eye position (top; shaded area, average ± s.d.), raster plots (centre) and the PETH (bottom) are shown. **h**, Same as in **f**, but for TTX-blinded animals. Left, *n* = 97; right, *n* = 67 (8 mice). Green, example neuron in **g**. 0–90% rise time of saccades, 27 ms.

The response to saccades of V1 neurons may simply result from the saccade-induced motion of the image on the retina, as such motion induces direction-selective responses^18,19^. To determine whether V1 activity in response to saccades also contains a non-visual component, we performed recordings in head-fixed, awake mice with a computer monitor placed contralateral to the recorded hemisphere, as this configuration allowed us to control the visual environment of the animal more precisely (Fig. 2a,b).

**Extended Data Fig. 2:**
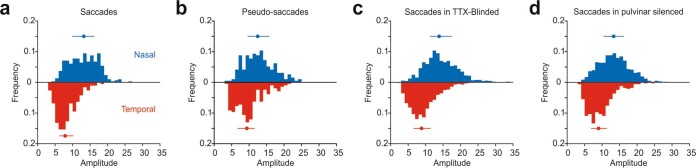
Distribution of real and pseudo-saccade amplitudes. a, Histogram of saccade amplitudes in control head-fixed mice. Blue, nasal saccades; red, temporal saccades. Bars indicate interquartile range, dot denotes median. Nasal saccades: median, 13.2; Q_1_, 9.9; Q_3_, 16.2; *n* = 296. Temporal saccades: median 7.9; Q_1_, 6.2; Q_3_, 10.26; temporal, *n* = 189. 4 mice. b, Histogram of pseudo-saccade amplitudes presented to the mice. For each mouse, we only analyzed the response to those pseudo-saccades whose amplitudes matched the amplitudes of the real saccades performed by the animal during the experiment. Nasal pseudo-saccades: median, 12.5; Q_1_, 9.6; Q_3_, 15.8; *n* = 193. Temporal pseudo-saccades: median 9.4; Q_1_, 6.7; Q_3_, 11.6; *n* = 207. c, Same as in (a) but for TTX-blinded animals. Nasal saccades: median, 13.9; Q_1_, 11.2; Q_3_, 17.5; *n* = 887. Temporal saccades: median 8.8; Q_1_, 6.5; Q_3_, 11.5; temporal, *n* = 561. 8 mice. d, Same as in (a) but for animals with the pulvinar silenced. Nasal saccades: median, 13.3; Q_1_, 10.4; Q_3_, 16.3; *n* = 579. Temporal saccades: median 9.0; Q_1_, 6.9; Q_3_, 11.4; temporal, *n* = 329. 9 mice.

Saccades in head-fixed animals occurred almost exclusively along the horizontal axis in either the nasal or temporal direction, in line with previous reports^20,21^ (Fig. 2c,d), and had a frequency of 3.1 ± 0.9 saccades per minute (nasal, 1.9 ± 0.6; temporal, 1.1 ± 0.4), mean amplitude of 10.7° ± 1.2° (nasal, 12.2° ± 1.9°; temporal, 8.2° ± 1.0°; Extended Data Fig. 2) and mean 10–90% rise time of 22.1 ± 1.5 ms (nasal, 18.3 ± 1.8 ms; temporal, 28.6 ± 3.6 ms), resulting in an average speed of 390° ± 146° per second (nasal, 470° ± 182° per second; temporal, 306° ± 119° per second; all statistics average ± s.d. of 13 mice).

**Extended Data Fig. 3:**
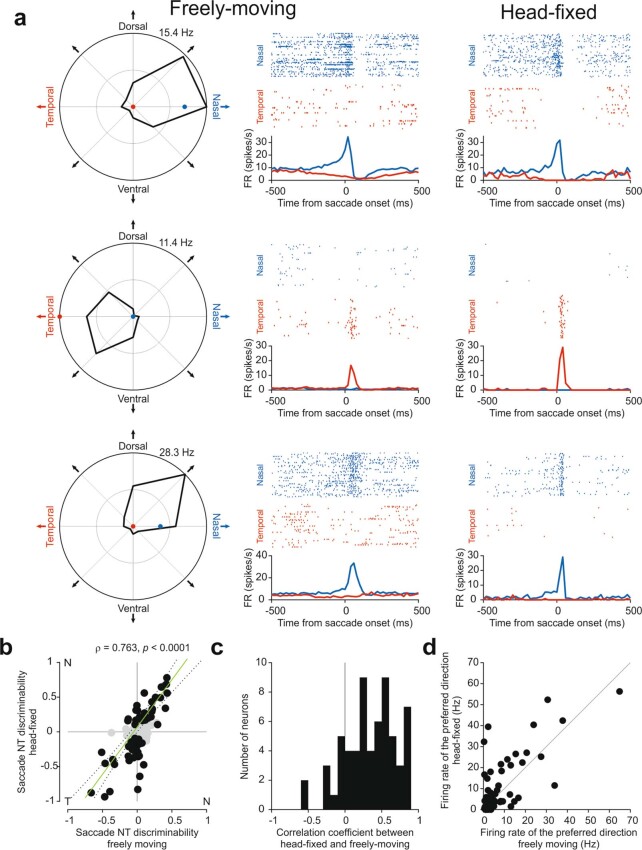
V1 neurons keep the same naso-temporal direction preference across freely-moving and head-fixed conditions. a, Comparisons of responses to nasal and temporal saccades under freely-moving condition and head-fixation for three example direction selective neurons. Left, polar plots showing saccade direction preferences under freely-moving conditions. The blue and red dots show the firing rate in response to nasal and temporal saccades, for the same neurons, under head-fixed conditions. Center, raster plots and PETH for nasal and temporal saccades under freely-moving conditions. Right, raster plots and PETH for nasal and temporal saccades under head-fixation. Note similar direction preference and response dynamics. The number of nasal and temporal saccades shown in the raster plots under head-free condition is matched to the number of nasal and temporal saccades in the head-fixed condition. The PETH under head-free condition is calculated from all nasal and temporal saccades recorded in this condition. b, Scatter plot of naso-temporal (NT) discriminability for saccades under freely-moving and head-fixed conditions, for all saccade responsive neurons under head-fixation. NT discriminability is a measure of how well an ideal observer can distinguish between the nasal and the temporal direction of saccades based on spike counts (negative and positive values indicate temporal and nasal preference, respectively; 0, no preference; see Methods). Black data points represent neurons that discriminate naso-temporal direction under head-fixed conditions. Pearson ρ = 0.76, *p* < 0.0001, *n* = 120 neurons, 5 mice. c, Histogram of correlation coefficients between PETHs of freely-moving and head-fixed conditions (−500 ms to +500 ms around saccade onset, FR calculated in 20-ms bins), for neurons that discriminate naso-temporal direction under head-fixed conditions. Coefficients were calculated for the preferred direction as determined under head-fixed conditions. d, Scatter plot of the response to preferred direction of saccades in freely-moving and head-fixed conditions (average firing rate in the first 100 ms window after saccade onset), calculated for neurons that discriminate naso-temporal direction under head-fixed conditions. Preferred direction as determined under head-fixed conditions.

**Extended Data Fig. 4:**
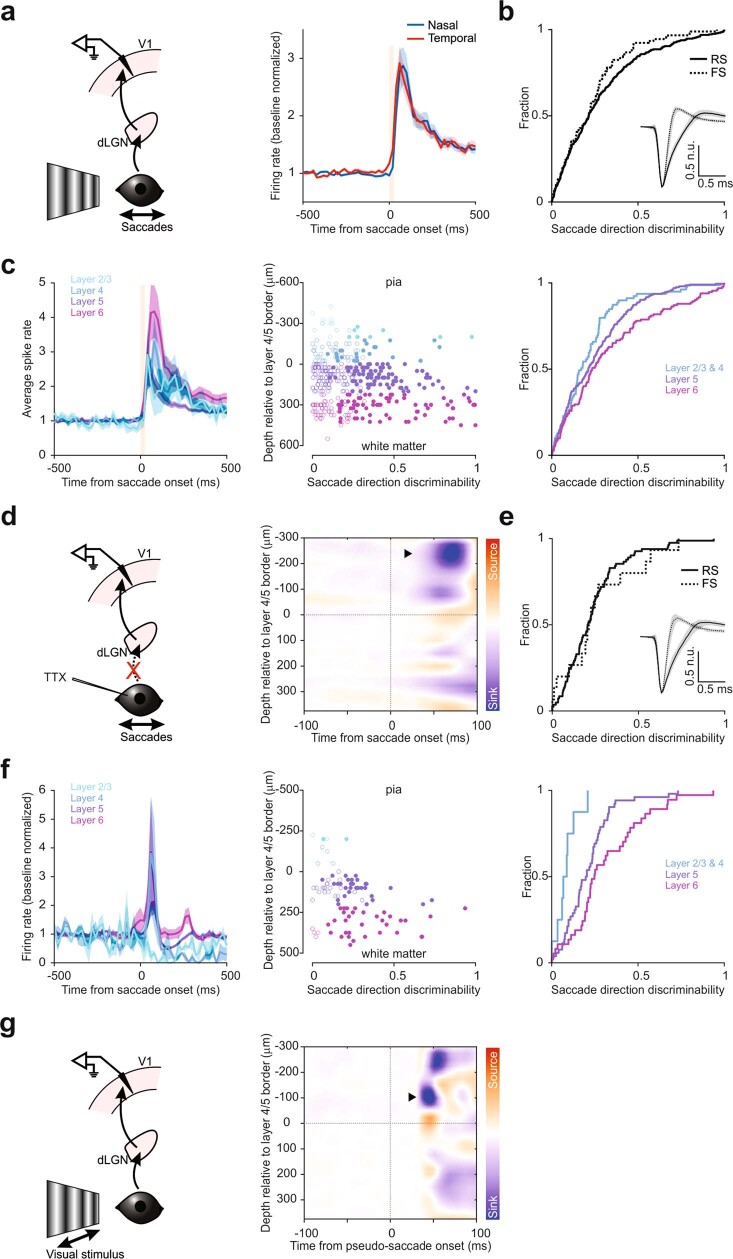
Saccade response across cortical depth and neuron class in control and TTX-blinded mice. a, Left, schematic of V1 recording during saccades on a vertical grating. Right, average PETH of saccade responsive neurons to nasal and temporal saccades. Baseline normalized. Shaded area, average ± s.e.m. Vertical orange bar, 0–90% rise time of saccades for reference (26 ms). Note the similarity of the population averaged response to nasal and temporal saccades. b, Cumulative frequency distribution (CFD) of direction discriminability (see Methods), plotted for saccade responsive regular-spiking (RS) and fast-spiking (FS) neurons. Overall, 53% (324 out of 607) and 82% (91 out of 111) of RS and FS neurons, respectively, responded to saccades. Anderson-Darling test, *p* = 0.29, *n* = 607 for RS, 111 for FS, 13 mice. Inset, median spike shape normalized to the trough. Shaded area, interquartile range. c, Left, average PETH of saccade responsive V1 neurons, sorted by cortical depth. All nasal and temporal saccades are included. Layer 2/3, 21 responsive neurons out of 54; layer 4, 58 out of 121; layer 5, 204 out of 334; layer 6, 132 out of 209. Baseline normalized. Shaded area, average ± s.e.m. Vertical orange bar, 0–90% rise time of saccades for reference (26 ms). Center, scatter plot of direction discriminability (x-axis) of all units as a function of cortical depth (y-axis). Open circles, statistically non-significant; filled, significant (see Methods). Color code as in left. Right, CFD of discriminability by cortical depth. Layers 2/3 and 4 were grouped together. 3-sample Anderson-Darling test, *p* < 0.0001. d, Left, schematic of V1 recording during saccades in TTX-blinded animals. Right, heat map of the current source density (CSD) analysis from an example animal. Note major sink in the supragranular layers (arrowhead). Data from 89 nasal and 82 temporal saccades. e, Cumulative frequency distribution (CFD) of saccade direction discriminability, plotted for saccade responsive RS and FS neurons in TTX-blinded animals. Overall, 42% (82 out of 194) and 48% (15 out of 31) of RS and FS neurons, respectively, responded to saccades. Anderson-Darling test, *p* = 0.313, *n* = 194 for RS, 31 for FS. Inset, median spike shape normalized to the trough. Shaded area, interquartile range. f, Left, average PETH of saccade responsive V1 neurons in TTX-blinded animals, sorted by cortical depth. All nasal and temporal saccades are included. Layer 2/3, 4 responsive neurons out of 13; layer 4, 4 out of 28; layer 5, 52 out of 119; layer 6, 37 out of 65. Shaded area, average ± s.e.m. Center, scatter plot of direction discriminability of all neurons (x-axis) as a function of cortical depth (y-axis). Open circles, statistically non-significant; filled, significant (see Methods). Color code as in left. Right, CFD of discriminability by cortical depth. Layers 2/3 and 4 were grouped together. 3-sample Anderson-Darling test, *p* = 0.0005. g, Same as in (d), but for visual stimuli in a control animal. The visual stimuli used here were pseudo-saccades, i.e., quick shifts of the grating that mimic visual scene changes induced by real saccades (see Main and Methods). Note early sink in layer 4 (arrowhead). Color scale same as in (d).

We validated that the properties of the V1 response to saccades recorded in freely moving animals were preserved under head fixation. First, we compared the saccade direction preference of individual V1 neurons recorded both in the head-free condition in the arena and in the head-fixed condition in front of a stationary vertical grating continuously displayed on the computer monitor. We analysed the response of these V1 neurons to nasal and temporal saccades (the predominant saccade directions occurring in head-fixed conditions; see above), irrespective of their preferred saccade direction observed in head-free conditions. In head-fixed conditions, the direction preference of V1 neurons clearly matched their preference in head-free conditions (Extended Data Fig. 3). Thus, individual neurons maintain their saccade direction preference irrespective of recording conditions. Second, the population response of V1 neurons to saccades under head fixation was similar to the response recorded in head-free conditions. The activity of the majority of V1 neurons (~58%) sampled across all layers was significantly modulated within the first 100 ms following saccade onset (415 of 718 neurons, 13 mice; Methods), and about half of the responding neurons exhibited a significant direction preference for either nasal or temporal saccades (192 of 415; Fig. 2e,f). Furthermore, again similarly to those in freely moving animals, the activity and directional preference both preceded and outlasted saccade duration by several tens of milliseconds, with saccades in the non-preferred direction resulting in suppression below baseline (Fig. 2f, right). The direction preference was observed in regular-spiking (putative excitatory) as well as fast-spiking (putative inhibitory) neurons (Extended Data Fig. 4). Thus, these data show that, under head-fixed conditions, the direction preference and response dynamics of V1 neurons to saccades are preserved, validating this experimental configuration to study the logic of responses to saccades in V1.

Two observations suggest that the response to saccades in V1 neurons may not be exclusively mediated by the shift of the image on the retina: the response starts before saccade onset, and fast-spiking neurons show strong direction discriminability (fast-spiking neurons have poor discriminability of visual stimulus direction^22,23^; Extended Data Fig. 4). To reveal the presence of a putative non-visual component in the response of V1 to saccades, we injected tetrodotoxin (TTX) in both eyes to block retinal activity. The distribution of saccade amplitudes was only weakly affected by TTX injection compared with control (Extended Data Fig. 2). Despite the complete block of visual input, saccades still triggered strong, directionally selective responses in V1 (Fig. 2g). In TTX-blinded animals, about half of the neurons in V1 responded to saccades, of which 69% discriminated the direction (97 responsive, 67 discriminating of 225 total, 8 mice; Fig. 2h), and the PETH for preferred and non-preferred directions diverged well before saccade onset (200-ms window before onset; evoked firing rate (FR)  ±  s.e.m.: 1.0 ± 0.3 Hz for preferred direction, −0.4 ± 0.2 Hz for non-preferred direction; Wilcoxon signed-rank test, one tailed: *P* = 3.3 × 10^−4^, *n* = 67; Fig. 2h, right). These data thus demonstrate the presence of a strong non-visual component in the response of V1 to saccades.

Interestingly, the impact of saccades on neuronal activity was layer dependent, showing a gradient of increasing excitability and discriminability as a function of depth (Extended Data Fig. 4). The complete block of visual responses with TTX allowed us to estimate the site of entry of the non-visual input by performing a current source density analysis of the saccade-triggered local field potential (LFP). The analysis identified a sink in the supragranular layers of V1, distinct from the initial sink in layer 4, in response to a visual input (Extended Data Fig. 4). Thus, mouse V1 receives a non-visual input that targets the superficial layers and that carries saccade direction information.

## Visual versus saccade direction preference

**Fig. 3:**
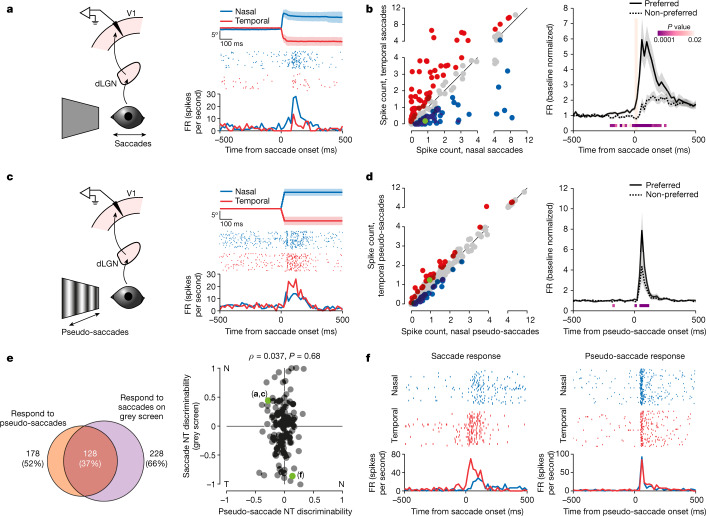
Direction preferences for saccades and visual motion are not correlated. **a**, Left, schematic of V1 recording during saccades on a grey screen. Right, example neuron preferring nasal saccades. Average eye position (top; shaded area, average ± s.d.), raster plots (centre) and the PETH (bottom) are shown. **b**, Left, scatterplot of the response to nasal and temporal saccades for all responsive neurons (*n* = 171, 4 mice). Blue, nasal preference; red, temporal preference; grey, no statistical difference; green, example in **a**. Right, average PETH of discriminating neurons for preferred and non-preferred saccade directions (*n* = 107 neurons, 4 mice). Shaded area, average ± s.e.m. Orange bar, 0–90% rise time of saccades (25 ms). *P* values are from the comparison of activity for preferred and non-preferred saccade directions (Wilcoxon signed-rank test, one tailed). **c**, Left, schematic of V1 recording during pseudo-saccades. Right, same example neuron as in **a**. This neuron prefers the temporal direction for pseudo-saccades (otherwise as in **a**). **d**, Left, scatterplot of the response to nasal and temporal pseudo-saccades (*n* = 582 neurons, 13 mice, including the 4 mice in **b**). Blue, nasal preference; red, temporal preference; grey, no statistical difference; green, example in **c**. Right, average PETH of discriminating neurons (*n* = 65 neurons; otherwise as in **b**). **e**, Left, Venn diagram of the number of neurons that respond to pseudo-saccades, saccades on a grey screen and both. Percentages are out of the entire population; based on four mice from **b** and **d** in which the responses to both saccades on a grey screen and pseudo-saccades were tested. Right, scatterplot of NT discriminability for pseudo-saccades (*x* axis) against saccades on a grey screen (*y* axis), for neurons that respond to both (128 neurons in **e**). *n* = 128 neurons, 4 mice. NT discriminability reports how well an ideal observer distinguishes between nasal and temporal saccades on the basis of spike counts (negative, temporal preference; positive, nasal preference; 0, no preference;  Methods). For this analysis, amplitudes and directions for pseudo-saccades were matched to those of real saccades on the grey screen. Green, example neurons in **a**, **c** and **f**. **f**, Example neuron showing altered direction preference for real saccades (left) and pseudo-saccades (right). Top, raster plots; bottom, PETHs.

The above results suggest that, during saccades on a grating, V1 receives both a non-visual input and a visual input triggered by the shift of the image on the retina. We thus determined, in individual V1 neurons, the relationship between their preference for saccade direction, imparted by the non-visual input, and their preference for the direction of the visual stimulus moving on the retina. To this end, we used pseudo-saccades, shifts of a vertical grating on the monitor designed to approximate the shifts on the retina resulting from real saccades. We compared the response of V1 to pseudo-saccades, the visual input, with that to saccades on a grey screen, the non-visual input (Fig. 3). The grey screen covered a large portion of the visual field (that is, a visual scene where the luminance is homogeneous in space; 128° in azimuth, 97° in elevation;  Methods and Fig. 3a,b), thus minimizing changes in retinal activity during saccades. Pseudo-saccades had a rise time of 25 ms and amplitudes (that is, horizontal shift of the grating) ranging from 3.0° to 24.6° (Fig. 3c,d and Methods). Shifts of the grating in the nasal direction were termed temporal pseudo-saccades because they generated a shift of the image on the retina in the same direction as that generated by real temporal saccades. Conversely, temporal shifts were termed nasal pseudo-saccades. For the analysis (Fig. 3e), we selected pseudo-saccades whose amplitudes and directions were matched to those of the real saccades performed by each animal during the recording session (average amplitude, 10.5° ± 1.1° (nasal, 12.1° ± 1.8°; temporal, 7.7° ± 1.0°; Extended Data Fig. 2); average speed, 420° ± 44° per second (nasal, 484° ± 70° per second; temporal, 307° ± 39° per second; all statistics average ± s.d. of 13 mice); Methods). Saccades on a grey screen elicited a response in 66% of V1 neurons (228 of 345, 4 mice). In line with the presence of a directionally selective non-visual input in V1 during saccades (Fig. 2), the response to saccades on a grey screen showed direction preference (145 of 228 responsive neurons) and both preceded and outlasted saccade duration by several tens of milliseconds (Fig. 3a,b). Pseudo-saccades elicited a response in a large fraction of V1 neurons (77%, 582 of 759, 13 mice), and 11% of responsive neurons (65 of 582) showed a preference for the nasal or temporal direction (Fig. 3c,d). In contrast to saccades on a grey screen or on a grating (Figs. 2e,f and  3a,b), the response to pseudo-saccades obviously did not precede the onset of the stimulus and there was no suppression of the average response to pseudo-saccades in the non-preferred direction (Fig. 3d, right). Many neurons that responded to pseudo-saccades also responded to real saccades on a grey screen (72%, 128 of 178, 4 mice; Fig. 3e, left). Notably, however, there was no correlation between the direction preference of neurons to pseudo-saccades and saccades on a grey screen, indicating that the direction preferences imparted by the visual and non-visual inputs to V1 neurons are independent (Pearson’s *ρ* = 0.037, *P* = 0.68, *n* = 128; Fig. 3e, right). The response of V1 neurons to saccades on a grating may thus result from the combination of the visual and non-visual inputs, whose direction preferences are uncorrelated. To test this hypothesis, we proceeded to identify the source of the non-visual input. Silencing this source should allow us to determine how the visual and non-visual inputs are combined in V1 during a saccade.

## Pulvinar origin of saccade input

**Extended Data Fig. 5:**
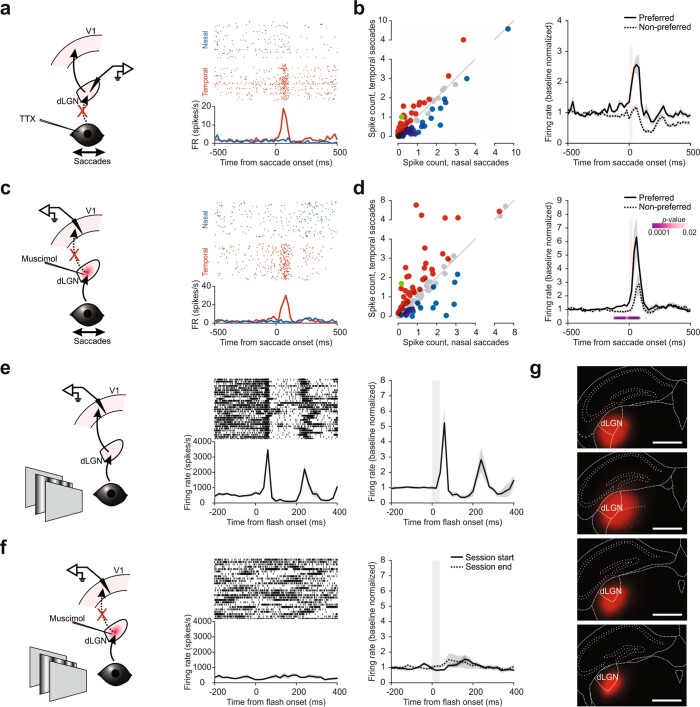
The dLGN is not the source of non-visual saccade input to V1. a, Left, schematic of dLGN recording during saccades in TTX-blinded animals. Right, example neuron preferring temporal saccades. Raster plots (top) and PETH (bottom). b, Left, scatter plot of the response to nasal and temporal saccades (average spike count in a 100 ms window from saccade onset) for all responsive dLGN neurons (*n* = 108, 4 mice). Blue, prefer nasal saccades; red, prefer temporal saccades; gray, no statistical difference; green, example neuron in (a). Right, average PETH for preferred and non-preferred saccade directions (n = 77 neurons, 4 mice). Baseline normalized. Shaded area, average ± s.e.m. Vertical orange bar, 0–90% rise time of saccades (26 ms). c, Left, schematic of V1 recording during saccades under dLGN silencing. Right, example neuron preferring temporal saccades. Raster plots (top) and PETH (bottom). d, Left, scatter plot of the response to nasal and temporal saccades (average spike count in a 100 ms window from saccade onset) for all responsive V1 neurons (n = 125, 4 mice). Blue, prefer nasal saccades; red, prefer temporal saccades; gray, no statistical difference; green, example neuron in (c). Right, average PETH for preferred and non-preferred saccade directions (*n* = 64 neurons, 4 mice). Shaded area, average ± s.e.m. Vertical orange bar, 0–90% rise time of saccades (26 ms). *P*-values, comparison of activity for preferred and non-preferred saccade directions in 20-ms bins (see Methods). Note the persistence of directionally selective saccade responses in V1 despite dLGN silencing. e, Left, schematic of V1 recording during a brief (32 ms) presentation of a full-field grating (see Methods). Center, multi-unit response from an example recording. Raster plot (top) and PETH (bottom). Right, average PETH of 3 mice. Baseline normalized. Shaded area, average ± s.e.m. 173.5 ± 29% average increase in evoked FR ± s.e.m. f, Left and center, same as in (a), but for mice injected with muscimol-BODIPY in the dLGN. Right, average PETH of 4 mice. Recording started after muscimol injection. Response was measured both at the start of the recording session and at the end. Note the lack of visual response in both cases. Baseline normalized. Shaded area, average ± s.e.m. Average increase in evoked FR ± s.e.m. was −16.7 ± 7.5% (start) and 14.1 ± 8.8% (end). g, Section images of the four mice injected with muscimol-BODIPY in the dLGN in (**b**). Red, BODIPY. Scale bar, 1 mm.

We recorded from the dorsolateral geniculate nucleus of the thalamus (dLGN), the main source of afferent visual information to V1, to determine whether it is also the source of the non-visual input, as neurons in this structure have previously been shown to respond to saccades^24–27^. In TTX-blinded animals, dLGN neurons responded to saccades, and their responses were selective for saccade direction (108 responsive, 77 discriminating, 198 total, 4 mice; Extended Data Fig. 5). To determine whether dLGN is the source of the non-visual input to V1, we silenced dLGN by muscimol injection in otherwise unmanipulated (that is, non-blinded) animals. In contrast to the lack of visual responses in V1 confirming efficient silencing of dLGN, V1 neurons still robustly responded to saccades and discriminated the two directions (125 responsive, 64 discriminating, 140 total, 4 mice; Extended Data Fig. 5). These data show that dLGN is not the main source of the non-visual saccade input to V1.

**Fig. 4:**
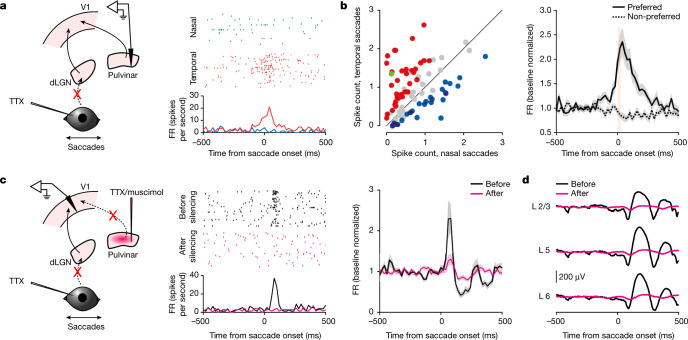
The pulvinar provides non-visual direction-selective saccade input to V1. **a**, Left, schematic of pulvinar recording during saccades in TTX-blinded animals. Right, example neuron preferring temporal saccades. Raster plots (top) and the PETH (bottom) are shown. **b**, Left, scatterplot of the response to nasal and temporal saccades for all responsive neurons (*n* = 84, 12 mice). Blue, nasal preference; red, temporal preference; grey, no statistical difference; green, example neuron in **a**. Right, average PETH of discriminating neurons (coloured data points on left scatterplot) for preferred and non-preferred directions (*n* = 61 neurons). Shaded area, average ± s.e.m. Orange bar, 0–90% rise time of saccades (26 ms). **c**, Left, schematic of V1 recording during saccades in TTX-blinded mice before and after pulvinar silencing. Centre, raster plot (top) and PETH (bottom) of an example neuron in response to nasal saccades before and after pulvinar silencing. Right, average PETH of saccade-responsive neurons before and after pulvinar silencing (*n* = 56 neurons, 5 mice). All nasal and temporal saccades are included. Shaded area, average ± s.e.m. **d**, LFPs from an example animal aligned to the time of saccades for layer 2/3 (L2/3), layer 5 (L5) and layer 6 (L6) before and after pulvinar silencing.

**Extended Data Fig. 6:**
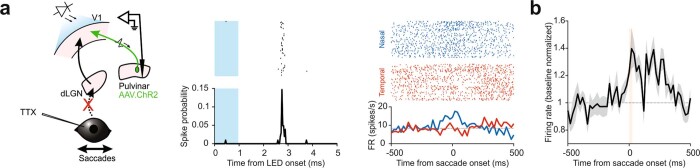
Pulvinar neurons that respond to saccades can project to V1. a, Left, Schematic of pulvinar recordings during saccades in TTX-blinded animal and optogenetic antidromic activation of pulvinar projections to V1. Center, example of antidromically activated neuron. Raster plot (top) and spike probability (bottom; 0.05 ms bin). Blue shaded area indicates time of the 1-ms LED illumination. Right, response of neuron shown in center panel to saccades. This neuron prefers nasal saccades. Raster plot (top) and PETH (bottom). Dotted line in PETH, baseline firing rate. b, Average PETH of saccade-responsive pulvinar neurons that were antidromically activated by illumination of V1 (*n* = 13 neurons, 3 mice). Shaded area, average ± s.e.m. Vertical orange bar, 0–90% rise time of saccades (26 ms).

**Extended Data Fig. 7:**
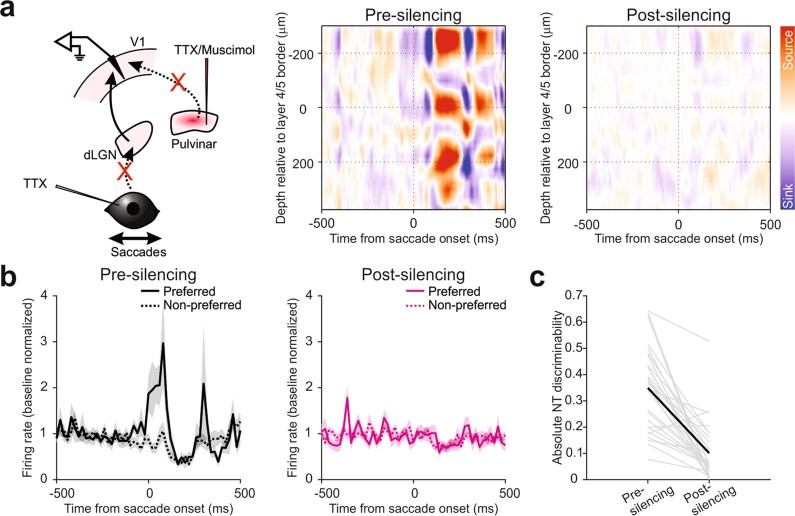
Silencing the pulvinar eliminates saccade response in V1 of TTX-blinded mice. a, Left, schematic of V1 recording during saccades in TTX-blinded animals before and after pulvinar silencing. Center, heat map of the current source density (CSD) analysis of an example animal, prior to pulvinar silencing. All nasal and temporal saccades are included. Note the strong sink in the superficial layers. Right, CSD heat map of the same animal, but after the pulvinar silencing. Color scale: same as in the center panel. Note the attenuated sink. b, Left, average PETH of discriminating neurons for preferred and non-preferred directions, prior to pulvinar silencing. Right, average PETH of the same neurons, but after pulvinar silencing. *n* = 29, 5 mice. Shaded area, average ± s.e.m. c, Discriminability of the 29 neurons in (b), pre and post silencing of the pulvinar. Gray, individual animals; black, average.

We next focused on the pulvinar, a higher-order thalamic nucleus with extensive projections to superficial layers of V1 (ref. ^28^), in line with the estimated entry point of the non-visual input (see above), and a structure in which neurons have also been shown to respond to saccades^27,29,30^. Recordings in the pulvinar in TTX-blinded animals showed that about a third of the neurons responded to saccades (84 of 245, 12 mice), many of which were also direction selective (61 of 84; Fig. 4a,b). Furthermore, the PETH of directionally selective pulvinar neurons for preferred and non-preferred directions diverged before saccade onset (200-ms window before onset; FR  ±  s.e.m.: 1.2 ± 0.4 Hz for the preferred direction, 1.0 ± 0.3 Hz for the non-preferred direction; Wilcoxon signed-rank test, one tailed: *P* = 0.005, *n* = 61; Fig. 4b, right). We also verified the presence of direct projections from these neurons to V1, using channelrhodopsin-2-mediated antidromic activation^31–33^ (Methods). Of 23 neurons that were identified in such a manner, more than half responded to saccades (13 of 23, 3 mice) and 5 neurons discriminated saccade direction (Extended Data Fig. 6). To determine whether neurons in the pulvinar provide the non-visual saccadic input to V1, we silenced the pulvinar (using either TTX or muscimol) while recording from V1 in TTX-blinded animals. Notably, pulvinar silencing abolished both the non-visual saccadic response in V1 neurons and the saccade-triggered LFP (88% ± 19% average decrease ± s.e.m. in saccade-evoked FR; Wilcoxon signed-rank test, one tailed: *P* = 0.012, *n* = 56; based on 56 saccade-responsive neurons of 140 pre-silencing neurons, 5 mice; Fig. 4c,d and Extended Data Fig. 7). Silencing the pulvinar also strongly reduced the ability of V1 neurons to discriminate saccade directions (before silencing, 6.8 ± 1.6 Hz difference in evoked FR ± s.e.m. between preferred and non-preferred directions; after silencing, 1.3 ± 0.6 Hz, 74% ± 11% reduction; Wilcoxon signed-rank test, one tailed: *P* < 0.0001, *n* = 29; based on 29 neurons that discriminated before silencing, 5 mice; Extended Data Fig. 7). Taken together, these results demonstrate that the pulvinar is the main source of the non-visual saccade response in V1.

## Isolating visual input during saccades

**Fig. 5:**
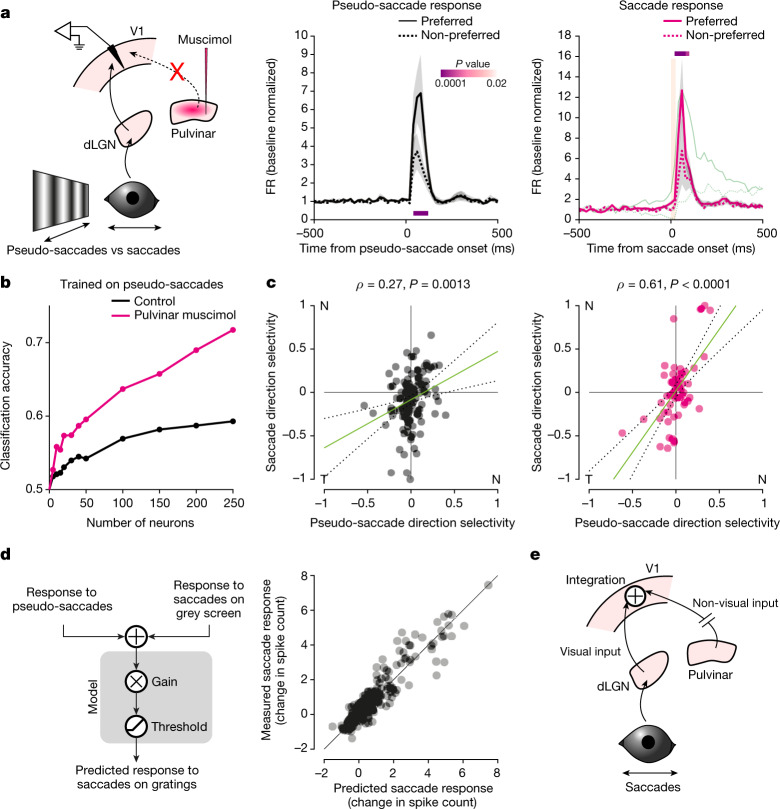
Non-visual and visual inputs are combined in V1 during saccades. **a**, Left, schematic of V1 recording during saccades on a grating while silencing the pulvinar. Centre, average PETH for pseudo-saccades recorded before pulvinar silencing (*n* = 34 neurons that discriminate pseudo-saccade direction out of 328 neurons, 9 mice). Right, average PETH for real saccades following pulvinar silencing (*n* = 34 neurons that discriminate saccade direction). Orange bar, 0–90% rise time of saccades (25 ms). Shaded area, average ± s.e.m. *P* values are from comparison of activity for preferred and non-preferred saccade directions in 20-ms bins (Wilcoxon signed-rank test, one tailed). Green trace, average PETH for saccades in control conditions for comparison of the time course (from Fig. 2f; scaled to the peak of response to the preferred direction). Following pulvinar silencing, the time course of the response to saccades resembles that of the response to pseudo-saccades, including a lack of separation before saccade onset. **b**, Classification accuracy for direction of motion (nasal or temporal) for a classifier trained on pseudo-saccades under the control condition and tested on real saccades under the control condition (black) or under pulvinar silencing (magenta), as a function of the number of neurons included in the analysis. **c**, Left, scatterplot of NT discriminability for real and pseudo-saccades for neurons that contribute to the classifier’s ability to discriminate (top 20% of neurons). Green line, linear regression (coefficient, 0.61; *P* = 0.0003); dotted lines, 95% confidence interval. *n* = 135 neurons, 13 mice. Right, same as to left, but real saccade responses were acquired after pulvinar silencing. Green line, linear regression (coefficient, 1.52; *P* < 0.0001). *n* = 61 neurons, 9 mice. Note the improved correlation. **d**, Left, schematic of the linear model used to predict the number of spikes evoked by saccades on a vertical grating on the basis of the response of neurons to pseudo-saccades and to saccades on a grey screen (Methods). Right, predicted number of spikes (*x* axis) plotted against the observed values (*y* axis). **e**, Integration of saccade direction-selective non-visual input from the pulvinar with saccade-induced visual motion alters the stimulus direction preference of V1 neurons during saccades.

The anatomical separation between the sources of the visual (dLGN) and non-visual (pulvinar) inputs to V1 provided us with the experimental opportunity to silence the non-visual input while sparing the visual input. Indeed, following silencing of the pulvinar, saccades on a grating evoked activity in V1 that was very similar to the activity evoked by pseudo-saccades, as shown by the average PETH (Fig. 5a). By contrast, under control conditions, the PETHs of the response to pseudo-saccades and to saccades on a grating showed very different dynamics, as illustrated above (compare Fig. 2f, right, to Fig. 3d, right). Under pulvinar silencing, the response to saccades no longer preceded saccade onset; that is, there was no separation between the preferred and non-preferred directions before saccade onset, and the time course resembled that of the response to pseudo-saccades. Moreover, saccades in the non-preferred direction resulted in an increase in firing rate rather than a decrease, similarly to pseudo-saccades in the non-preferred direction. These data show that, by silencing the pulvinar, we are able to isolate the visual inputs in V1 during saccades.

Silencing of the pulvinar allowed us to test directly whether the non-visual input enables individual V1 neurons to differentially respond to the same shift of the image on the retina depending on whether the shift was externally or self-generated. Given that V1 neurons receive both visual and non-visual inputs that impart direction preferences that are uncorrelated (Fig. 3e), we expect distinct patterns of activity in V1 in response to pseudo-saccades and saccades on a grating, even though they induce similar shifts of the image on the retina. Furthermore, we expect the patterns to become more similar to each other following silencing of the pulvinar (that is, after isolating the visual input). To investigate the similarity of the patterns of activity evoked by pseudo-saccades and saccades on a grating, we trained a classifier to distinguish the direction of pseudo-saccades on the basis of the population activity of V1 neurons and tested it on the response to real saccades on a grating either in control conditions or after pulvinar silencing (Methods). The accuracy of the classifier provides a metric for the similarity in activity patterns between pseudo-saccades and real saccades. The classifier performed much better at distinguishing the direction of real saccades when the pulvinar was silenced than under control conditions (Fig. 5b). Thus, the data show that saccades on a grating and pseudo-saccades induce distinct patterns of activity in V1 despite similar shifts of the image on the retina. The patterns become similar following silencing of the pulvinar.

**Extended Data Fig. 8:**
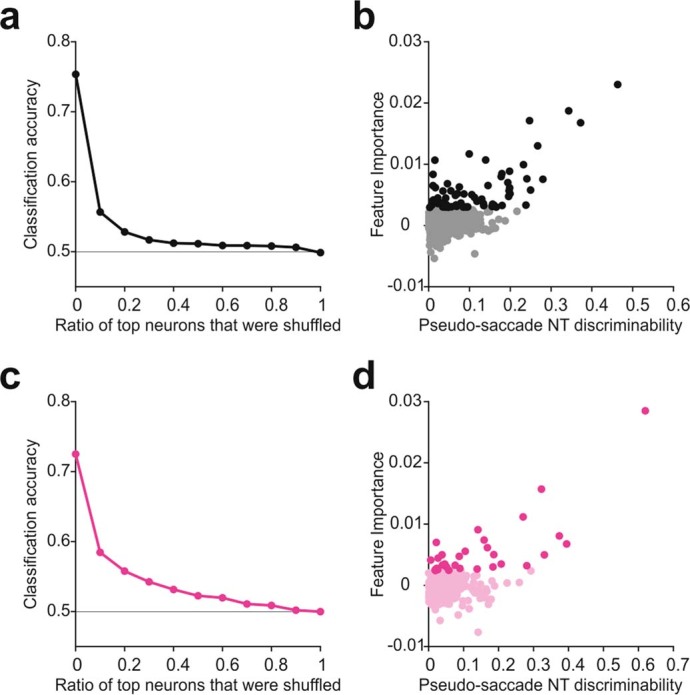
Identification of V1 neurons that contribute to the performance of the classifier of pseudo-saccade direction. a, Classification accuracy of pseudo-saccades (cross-validated 10-fold) plotted against the ratio of top contributing neurons from control animals (*n* = 13 mice) whose pseudo-saccade responses were shuffled in the training dataset. The neurons were first ranked by their contribution to the accuracy of the classifier in decoding pseudo-saccade direction, determined from permutation feature importance (see Methods). b, The discriminability of pseudo-saccade direction (x-axis) plotted against each neuron’s contribution to the accuracy of the classifier (y-axis, feature importance). Darker shade, top 10% of the contributing neurons. c-d, Same as in (a-b), but for animals in which the pulvinar was later silenced (*n* = 9 mice). Note that the responses to pseudo-saccades used here were collected prior to the silencing.

To determine whether the improved performance of the classifier was due to a better correlation of direction preference between pseudo-saccades and real saccades, we proceeded as follows: we focused on the top 20% of neurons ranked by their contribution to the accuracy of the classifier in decoding pseudo-saccade direction. The response of this population to pseudo-saccades contained most of the information about pseudo-saccade direction, as excluding this population from the classifier resulted in close to chance performance (Extended Data Fig. 8). Furthermore, as expected, the ranking correlated well with pseudo-saccade direction discriminability (Extended Data Fig. 8). Under control conditions, the direction preferences of these neurons to real saccades and pseudo-saccades was poorly correlated. This is in line with the poor performance of the classifier in inferring real saccade direction when trained on pseudo-saccades (Fig. 5c, left). However, following pulvinar silencing, the correlation between the direction preferences of real saccades and pseudo-saccades increased significantly (*P* = 0.0031, *z*-test after Fisher’s *z* transformation, one tailed; Fig. 5c, right). Thus, after silencing of the pulvinar, the response of V1 neurons to saccades is mainly driven by the visual input, explaining the increase in the classifier’s performance.

**Extended Data Fig. 9:**
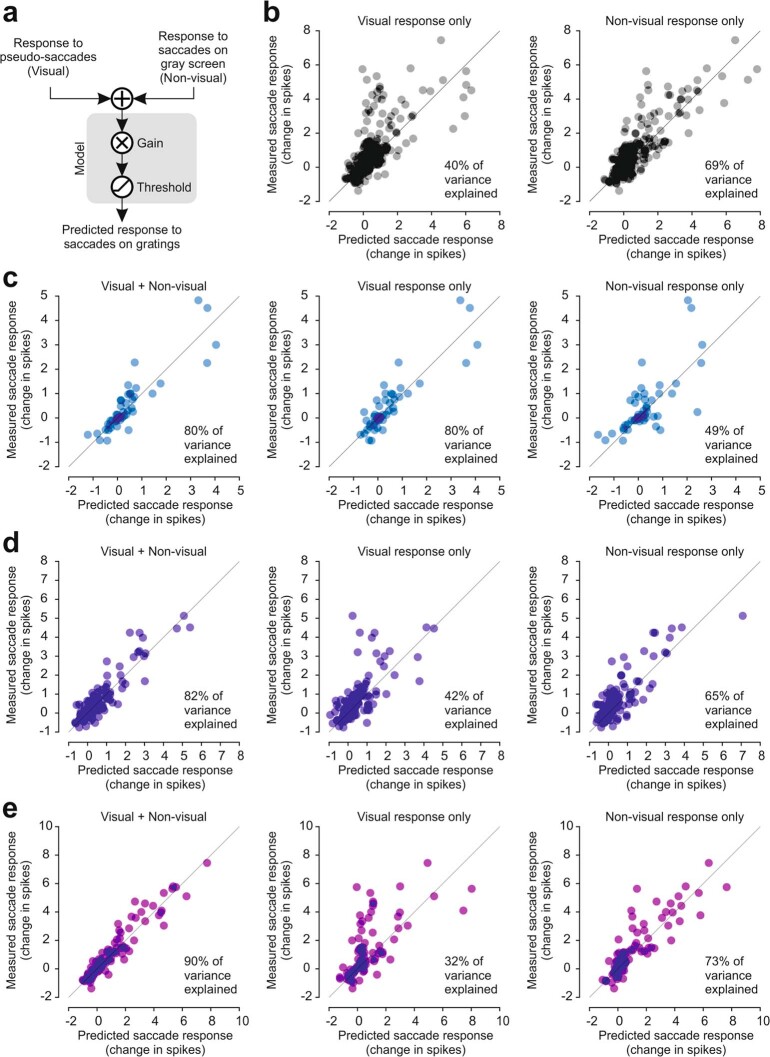
The visual and the non-visual components of the V1 response to saccades each capture only part of the V1 response to saccades on a grating. a, Schematic of the linear regression-based model used to predict the number of spikes evoked by saccades on a vertical grating. The model is based on the response of neurons to pseudo-saccades (visual component) and to saccades on a gray screen (non-visual component). Results from the sum of the two inputs are shown in Fig. 5g in which the model explains 86% of the observed variance. b, Model prediction results for all neurons that respond to pseudo-saccades and saccades on a gray monitor. Left, predicted number of spikes from the response to pseudo-saccades alone (x-axis) plotted against the observed values (y-axis). This model explains only 40% of the observed variance (gain 0.97, *p* < 0.0001). Right, predicted number of spikes from the response to saccades on a gray screen alone (x-axis) plotted against the observed values (y-axis). This model explains only 69% of the observed variance (gain 0.89, *p* < 0.0001). c, Model prediction results for layer 2/3 and 4 neurons that respond to pseudo-saccades and saccades on a gray monitor. Left, predicted number of spikes from the summed response to pseudo-saccades and saccades on a gray monitor (x-axis) plotted against the observed values (y-axis). This model explains 80% of the observed variance (gain 0.49, *p* < 0.0001). Center, predicted number of spikes from the response to pseudo-saccades alone (x-axis) plotted against the observed values (y-axis). This model explains 80% of the observed variance (gain 0.62, *p* < 0.0001). Right, predicted number of spikes from the response to saccades on a gray screen alone (x-axis) plotted against the observed values (y-axis). This model explains only 49% of the observed variance (gain 1.49, *p* < 0.0001). Note the lack of difference in prediction accuracy between the summation model (left) and the visual response model (center). The response to saccades on a grating in layer 2/3 is mainly shaped by the visual inputs. d, Same as in (c), but for layer 5 neurons. Left, gain 0.63, *p* < 0.0001. Center, gain 1.22, *p* < 0.0001. Right, gain 0.85, *p* < 0.0001. Note the increase in prediction accuracy in the summation model (left) compared to the other models. The response to saccades on a grating in layer 5 is shaped by the combination of visual and non-visual inputs. e, Same as in (c), but for layer 6 neurons. Left, gain 0.66, *p* < 0.0001. Center, gain 1.17, *p* < 0.0001. Right, gain 0.88, *p* < 0.0001. Again, note the increase in prediction accuracy in the summation model (left) compared to the other models.

To assess how the visual and non-visual inputs are integrated in V1 neurons, we built a simple model using linear regression. We based this analysis on 128 neurons that responded both to saccades on a grey screen and to pseudo-saccades (four mice; Fig. 3e, left). Using a simple summation of the visual and non-visual inputs, this model explained 86% of the variance in the number of spikes induced by a saccade in front of a vertical grating (Fig. 5d; spikes counted within the first 100 ms following saccade onset; Methods). The estimated gain on the combined input was 0.62 (*P* < 0.0001), suggesting a linear integration with reduced gain. By contrast, when using the response from only visual input or from only non-visual input, the model explained 40% and 69% of the variance, respectively (Extended Data Fig. 9). Interestingly, the relative contributions of the visual and non-visual inputs showed a gradient across layers, with the response in deeper layers progressively shaped more by the non-visual input (Extended Data Fig. 9). Taken together, the data indicate that linear integration of the visual and non-visual inputs during saccades enables V1 to differentially represent the shift of images on the retina depending on whether they are generated by external motion or by a saccade.

## Discussion

Our study shows that the activity pattern to an image moving on the retina differs, as early as in V1, depending on whether that movement is generated by motion in the environment or by motion of the eyes. This is because, during saccades, V1 combines a non-visual input that originates from the pulvinar and that depends on saccade direction with the visual input originating from the retina. The combination of non-visual and visual inputs alters the stimulus direction preference of individual V1 neurons, owing to the fact that the direction preference to the non-visual input does not correlate with that to the visual input.

**Extended Data Fig. 10:**
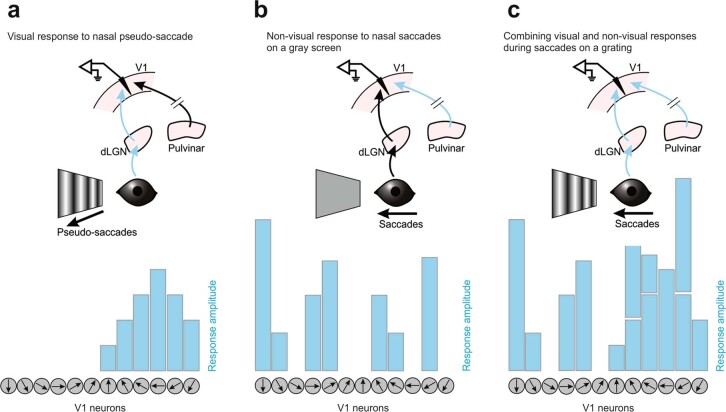
Integration of visual and non-visual inputs during saccades alters direction preference in V1. a, Illustration showing responses to a nasal pseudo-saccade (leftward in the schematic) in a population of 12 example V1 neurons, each tuned to a different direction of motion of a pseudo-saccade (gray circles, preferred direction indicated by arrows). A nasal pseudo-saccade activates, to various extents, six neurons whose preferred direction is close to the direction of motion of the pseudo-saccade. The other six neurons would have been activated by a temporal pseudo-saccade. A structure downstream of V1 responsible for determining the stimulus direction will count the V1 neurons “voting” for either nasal or temporal direction in order to establish the direction of the pseudo-saccade: Since more neurons voted nasal, this was most likely the direction of the pseudo-saccade. b, Illustration showing responses to a real nasal saccade on a gray screen (i.e. a situation in which V1 experiences only the non-visual input originating in the pulvinar), in the same 12 V1 neurons as in (a). The tuning preference of V1 neurons to the direction of the visual input are *not* matched with their tuning preference to the direction of the non-visual input. Thus, a nasal saccade on a gray screen activates a subset of V1 neurons that has no apparent relationship with the subset of neurons activated by the nasal pseudo-saccade in (a). c, Illustration showing responses to a real nasal saccade on a grating (i.e., a situation in which V1 experiences both the visual and the non-visual inputs), in the same 12 V1 neurons as in (a). In this situation, V1 experiences a combination (i.e., a summation, see Fig. 5d) of the visual and non-visual inputs. The pattern of activity of the population of V1 neurons in response to a real nasal saccade on a grating vastly differs from the pattern of activity generated by a nasal pseudo-saccade (a), thus “scrambling” the response. The downstream structure now counts both temporal and nasal votes and cannot accurately attribute a visual stimulus direction. Calculation of a confidence level for the direction (i.e. a metric that quantifies the similarity of a given V1 activity pattern to the prototypical V1 response patterns to pseudo-saccades in nasal or temporal direction) may prevent the decoder from reporting motion during saccades.

The combination of two directionally selective yet uncorrelated inputs during saccades presents a simple and effective strategy that enables mouse V1 to briefly reconfigure the representation of visual motion in each individual neuron independently, as if the direction selectivity of the population had been ‘scrambled’ (Extended Data Fig. 10). Interestingly, similar changes in direction preference have been reported in higher visual areas of primates around the time of saccades, possibly indicating conserved neuronal mechanisms^17,34^. The change in representation of self-generated stimuli may work in concert with previously reported mechanisms in which sensory responses to self-generated stimuli are suppressed^35–38^.

The dynamics of the V1 responses to saccades in freely moving and head-fixed animals were remarkably similar, warranting the use of head-fixed conditions to study the interaction between visual and non-visual inputs. In both freely moving and head-fixed animals, V1 activity was modulated before saccade onset, peaked shortly after the saccade, outlasted the saccade duration and showed marked saccade direction preference. These dynamics may reflect a motor command, reaching V1 as an efference copy or corollary discharge. We cannot exclude the possibility, however, that later parts of the non-visual saccade response in V1 may also reflect proprioceptive signals originating in the eye muscles. Because gaze shifts result from coordination of eye and head movements^5,6^, the V1 response around the time of saccades may reflect the combined representation of eye and head motor commands.

Our silencing experiments indicate that the pulvinar is the source of the non-visual saccade signals in V1. Indeed, following pulvinar silencing, the V1 responses to pseudo-saccades and real saccades on a grating became very similar, in terms of both dynamics and direction preference. The remaining difference in the responses may be due to incomplete silencing of the pulvinar. The pulvinar has been shown to represent movement-related activity^27, 29, 39^, possibly received as an efference copy through collateral branches of cortical neurons that project to brainstem motor nuclei^40,41^. Furthermore, it also receives substantial input from the superior colliculus^42–44^, a midbrain structure involved in saccade initiation^45^. While we have identified, in TTX-blinded animals, direct projections from saccade-responsive pulvinar neurons to V1, indirect routes also remain possible.

In humans, motion on the retina induced by saccades is often perceptually unnoticed, a phenomenon termed saccade omission^46^. If similar mechanisms to those identified here are at work in the human brain, the distinct cortical representation of self- and externally generated motion may prevent downstream areas from decoding the direction of visual motion induced by a person’s own saccadic eye movement. Despite the lack of perceptual experience, however, studies in humans also show that visual processing remains active during saccades^47,48^. These results are consistent with our finding that the visual signal, rather than being suppressed, is combined with the non-visual input. Furthermore, our model suggests that the gain of the visual signal is reduced during saccades, in line with the notion that a reduction in gain could contribute to saccade suppression^49^. Purely visual mechanisms that arise as early as in the retina may also contribute to saccade suppression^50^.

In conclusion, we have uncovered a circuit mechanism that allows V1 to distinguish motion induced by the animal’s own eye movement from changes in the environment through the combination of two independent inputs whose response properties are uncorrelated. This mechanism may represent a general strategy for sensory cortices to distinguish between self- and externally generated stimuli.

## Methods

### Mouse handling

Experiments were conducted in accordance with the regulations of the Institutional Animal Care and Use Committee of the University of California, San Diego, and of the University of California, San Francisco. All mice used in this study were wild-type C57BL/6J males or females from the Jackson Laboratory (JAX 000664) and were of postnatal ages of 3 to 6 months. No statistical methods were used to predetermine sample size. The experimenter was not blind to the experimental conditions.

Animals were familiarized to head fixation for at least 2 weeks before recording. During this time, they were also familiarized to visual stimuli that would be used during recording. Animals were head-fixed on a custom-made passive treadmill, either circular or linear, and were free to run.

### Eye tracking

Video-oculography was used to track the movement of the right eye in both freely moving and head-fixed mice, contralateral to the hemisphere in which recordings were conducted.

In freely moving mice, the right eye was tracked using a miniature camera (Arducam Noir Spy Camera) mounted on a custom-designed holder attached to the skull. The eye was illuminated using an infrared LED mounted on the holder. The video was acquired at 90 Hz through Raspberry Pi 3B+ using RPiCamera-Plugin^51^.

For head-fixed experiments, a high-speed camera (IMPERX, IPX-VGA-210-L) was fitted with a 45-mm extension tube, a 50-mm lens (Fujifilm, Fujinon HF50HA-1B) and an infrared pass filter (Edmund Optics, 65-796). Images were acquired at 200 Hz through a frame grabber (National Instrument, PCIe-1427). An infrared hot mirror (Edmund Optics, 43-958) was placed parallel to the antero-posterior axis of the animal (1 inch from the eye) in between the animal and the LCD monitor, and the camera captured the image of the eye through its reflection. The camera was angled at 59° relative to the antero-posterior axis. Three infrared 880-nm LED emitters (Digi-Key, PDI-E803) were used to illuminate the eye.

### Measuring the angular position of the eye

In head-fixed animals, one of the three infrared LEDs (see above) was aligned with the optical axis of the camera and served as a reference to calculate pupil position. The pupil was identified by thresholding and fitting an ellipse. We computed *α*, the angular position of the eye, according to sin(*α*) = *d*/*R*_p_, where *d* is the projected distance on the camera image between the centre of the ellipse and the corneal reflection (CR) of the reference LED and *R*_p_ is the length of the radius that connects the rotational centre of the eye with the centre of the pupil on the plane that harbours the pupil. Note that *R*_p_ is shorter than the radius of the eyeball. *R*_p_ was estimated before the experiments as follows: the camera, together with the reference LED, was swung by calibration angles *γ* of ±10° along a circumference centred on the rotational centre of the eye (more precisely, on the rotational centre of the mirror image of the eye, as the eye was imaged through an infrared mirror) such that the CR of the reference LED remained stationary relative to the image frame of the camera. We used different values of *d* obtained with different *γ* to estimate *R*_p_. Complicating the issue is the fact that *R*_p_ is not fixed but changes with the size of the pupil (that is, the distance from the rotational centre of the eye to the plane that harbours the pupil increases with constriction of the pupil^52^). We thus computed *R*_p_ under various luminance conditions to change pupil diameter (*D*_p_, the long axis of the fitted ellipse) and obtained the following linear relationship: *R*_p_ = *r* – *a* × *D*_p_, where *r* is the radius of the eyeball; *a* typically ranges between 0.05 and 0.25. During eye tracking in both freely moving and head-fixed animals, this relationship was used to determine *R*_p_ for every video frame on the basis of pupil diameter. In some mice, *R*_p_ was estimated using the relationship obtained from littermates or other similarly sized mice. The details of the eye tracking method during head fixation have been published previously^53,54^.

In freely moving mice, to delineate the pupil, eight points along the edge of the pupil were tracked post hoc using DeepLabCut^55^ and were fitted with an ellipse. The centre of the pupil was defined as the centre of the ellipse, and the centre of the projected eye on camera C (equivalent to CR in head-fixed mice; see above) was estimated by using the orientations of the ellipses at multiple pupil positions where *d* is the projected distance between C and the centre of the pupil. The angular position of the eye, *α*, was computed as in head-fixed animals according to sin(*α*) = *d*/*R*_p_. *R*_p_ was estimated from the equation *R*_p_ = *r* – *a* × *D*_p_ obtained under head fixation.

### Surgery

Mice were implanted with either a custom T-shaped head bar (head-fixed experiments) or three threaded screw inserts arranged in a triangle (head-fixed and freely moving experiments; McMaster-Carr, 92395A109). Implantation was done stereotactically using an inclinometer (Level Developments, DAS-30-R) connected to a USB I/O device (National Instruments, USB-6008), such that the axes of the electrode manipulators for acute, head-fixed recordings would be aligned to the antero-posterior, medio-lateral and dorso-ventral axes of the skull. Mice were anaesthetized with 1–1.5% isoflurane and kept on a feedback-regulated heating pad to maintain body temperature at 37 °C (FHC, 40-90-8D). Before surgery, mice were given buprenorphine subcutaneously. Before incision, topical lidocaine cream was applied to the skin. Once the scalp and fascia were removed, the head bar or the screw inserts were cemented using dental cement (Lang Dental, Ortho-Jet for head bars; 3M ESPE, Relyx Unicem2 for screw inserts). Animals were allowed to recover in their home cage for at least 1 week following surgery.

For mice prepared for freely moving experiments, an extracellular electrode (Diagnostic Biochips, P64-4) mounted on a custom-designed hat for chronic recording was implanted 1 d before the recording session using dental cement. This procedure was performed weeks after initial implantation of the screw inserts. Mice were anaesthetized with 1–1.5% isoflurane and kept on a feedback-regulated heating pad. The electrode held by a holder was lowered to 1,100 mm below the pia surface using micromanipulators, and the hat was cemented in place before retracting the holder. The cranial window over V1 was ~200 mm by ~200 mm and was covered with silicone gel after electrode insertion to prevent V1 from drying. A ground wire (A-M Systems) was inserted in the cerebellum. A custom-designed camera mount was also attached to the head using the previously implanted screw threads (see above).

In head-fixed experiments, cranial windows for extracellular recording were made 1 or 2 d before the recording sessions. For all recordings, the size was ~500 µm to 1 mm by ~500 µm to 1 mm. Whiskers that would interfere with eye tracking were also trimmed at this point. Following craniotomy, the window was sealed with biocompatible silicone sealant until the recording session (World Precision Instruments, Kwik-Cast). The cranial windows were centred around the following coordinates that were marked during head bar or screw insert implantation:

V1 recording: 2.7 mm lateral to the midline, 4.1 mm posterior to the bregma

Pulvinar recording: 1.2 mm lateral to the midline, 1.9 mm posterior to the bregma

dLGN recording: 2.4 mm lateral to the midline, 2.2 mm posterior to the bregma

For identification of pulvinar neurons that send projections to V1 through optogenetic antidromic activation, AAV2/1.hSyn.ChR2(H134R)-eYFP.WPRE.hGH (Addgene, 26973P) was injected into the pulvinar in the left hemisphere, before implantation of the head bar or screw heads.

### Visual stimulation

Visual stimuli were presented on an LCD monitor running at 240 Hz (Gigabyte, AORUS KD25F) to the right eye, contralateral to the hemisphere in which recordings were performed. The monitor was angled at 31° anticlockwise relative to the antero-posterior axis of the animal and tilted 20° towards the animal relative to the gravitational axis. It was positioned such that the tangent point between the plane of the monitor and a sphere around the centre of the eye was in the centre of the monitor. The distance from the centre of the eye to the tangent point was 133 mm, with the monitor covering 128° of the field of view horizontally and 97° vertically. In the experiment described in Fig. 2g (a full-field flash), an LCD monitor running at 75 Hz was used.

The static vertical grating used in the experiments described in Figs. 2 and 5 was a full-field sinusoidal grating with 70% contrast, a spatial frequency of 0.08 cycles per degree (cpd) and a mean luminance of 40–60 cd m^–2^ (gamma corrected; fixed luminance for each animal). It was spherically morphed around the centre of the animal’s right eye to maintain the same spatial frequency across different spatial locations on the retina. For pseudo-saccades, the exact same grating was quickly shifted horizontally once every 1.5 s on average, over the span of seven frames (six inter-frame intervals, 25 ms). The speed of the shift over the seven frames was linear. The direction and amplitude of each shift were predetermined by randomly drawing from the distribution of real saccades collected separately from wild-type unmanipulated mice. For a nasal pseudo-saccade, the grating was shifted in the temporal direction, and, for a temporal pseudo-saccade, the grating was shifted in the nasal direction. Post hoc, every pseudo-saccade was checked for display errors such as a dropped frame. All pseudo-saccades that occurred within 500 ms of a real saccade were also discarded from further analysis, which resulted in about 350 pseudo-saccades for each animal over a span of 10 min. We then resampled the pseudo-saccades to match the direction and amplitude of the real saccades collected from the same animal. To increase statistical power, we resampled two matching pseudo-saccade events for every saccade. The mean ± s.d. of the difference in amplitude between a real saccade and its matched pseudo-saccades was 0.18° ± 0.47° (446 pseudo-saccades, 4 mice) for the experiments in Fig. 3e and 0.18° ± 0.45° (942 pseudo-saccades, 9 mice) in Fig. 5a.

For every animal, response to pseudo-saccades was collected at the beginning of the experiment. Response to real saccades using the static grating was collected after the pseudo-saccade session. The two responses were collected separately, to maximize our chances of obtaining saccades whose responses were not contaminated by pseudo-saccade responses.

To verify the absence of visual responses, following either intraocular TTX injection or muscimol injection in dLGN, we used the following visual stimuli: for the intraocular TTX injections, we used a full-field luminance change from 0 cd m^–^^2^ to 100 cd m^–2^ lasting 26 ms. For muscimol injection in dLGN, we used a full-field vertical grating (0.02 cpd; contrast, 0.5), presented every 10 s for 32 ms and preceded and followed by a grey screen of the same average luminance of 40 cd m^–2^.

All visual stimulation protocols were custom written in LabVIEW (National Instruments) and MATLAB (Mathworks) using Psychophysics Toolbox 3 (refs ^56,57^).

### Acute extracellular recording in head-fixed mice

All recordings in this study were performed on the left hemisphere. On the day of recording, animals were first head-fixed and the Kwik-Cast sealant was gently removed. Artificial cerebrospinal fluid (140 mM NaCl, 2.5 mM KCl, 2.5 mM CaCl_2_, 1.3 mM MgSO_4_, 1.0 mM NaH_2_PO_4_, 20 mM HEPES and 11 mM glucose, adjusted to pH 7.4) was quickly applied to the craniotomy to prevent the exposed brain from drying. Different configurations of silicon probes were used over the course of the study: A2x32-5mm-25-200-177-A64 (NeuroNexus), A1x64-Poly2-6mm-23s-160-A64 (NeuroNexus), A1x32-Poly2-10mm-50s-177-A32 (NeuroNexus) and ASSY-77 H2 (Cambridge NeuroTech). Using a manipulator (Luigs & Neumann), the probes were slowly lowered to the recording site. Probes were lowered to 1,000 μm below the pia for V1, 3,000 μm below the pia for dLGN and 2,900 μm below the pia for the pulvinar. For recordings in the thalamus, the probes were painted with lipophilic DiI before insertion visualization of the recording track. Successful targeting was verified post hoc.

For optogenetic activation of the axon terminals of pulvinar neurons, a glass fibreoptic cable (960-μm core, NA = 0.63; Doric Lenses) connected to a 465-nm LED light source (Doric Lenses, LEDC1-B_FC) was placed ~500 μm above the craniotomy on V1. The light source was driven by an LED driver (Thorlabs, LEDD1B) at 1,000 mA for 1 ms every 6 s for 10 min (100 trials).

Recordings were started 15 min after insertion of the probes. Signals were sampled at 30 kS s^–1^ using 64 channel headstages (Intan Technologies, C3315) combined with adaptors (NeuroNexus, Adpt.A64-Omnetics32_2x-sm), connected to an RHD USB interface board (Intan Technologies, C3100). The interface board was also used to acquire signals from photodiodes (TAOS, TSL253R) placed on the visual stimulation monitor as well as TTL pulses used to trigger the eye tracking camera and the LED. These signals were used during analyses to synchronize visual stimulus timings, video acquisition timings and LED photostimulation timings with electrophysiological recordings. All raw data were stored for offline analyses. Occasionally, we recorded from the same animal on two successive days, provided no pharmacological manipulation was performed on the first day. In these instances, the craniotomy was resealed with Kwik-Cast after the first recording session. For post hoc histological analysis, brains were fixed in 4% paraformaldehyde (PFA) in PBS overnight at 4 °C.

### Extracellular recording in freely moving mice

Mice were habituated in an acrylic open-air recording chamber under ambient light (length × width × height = 13.25 inches × 9 inches × 9.5 inches) for 1 h each day for 3 d before the day of recording. On the day of recording, a miniature camera connected to Raspberry Pi (see ‘Eye tracking’) was mounted on the camera mount, and the implanted electrode was connected to an RHD USB interface board (Intan Technologies, C3100). The TTL pulses from Raspberry Pi, used to synchronize the video frames with the electrophysiological signals, were also acquired through the interface board. Each recording session was 90 min long.

### Pharmacology

Intraocular injection of TTX (40 μM) was performed 2 h before recording under isoflurane anaesthesia. A typical procedure lasted less than 5 min. Carbachol (0.011% (wt/vol)) was co-injected with TTX to prevent the pupil from fully dilating, as a fully dilated pupil reduces the accuracy of eye tracking. Immediately before the injection, a drop of proparacaine hydrochloride ophthalmic solution was applied to the eye as a local anaesthetic (Bausch + Lomb; 0.5%). TTX solution was injected intravitreally using a bevelled glass micropipette (tip diameter, ~50 μm) on a microinjector (Nanoject II, Drummond) mounted on a manual manipulator. One microlitre was injected in each eye, at a speed of 46 nl s^–1^. In some animals, the injection solution also contained NBQX (2,3-dioxo-6-nitro-7-sulfamoyl-benzo[f]quinoxaline; 100 μM) and APV ((2*R*)-amino-5-phosphonovaleric acid; 100 μM). The animals were head-fixed for recording following a 2-h recovery period in their home cage. Suppression of retinal activity was confirmed for every experiment by a lack of response in visual cortex to a full-field flash of the LCD monitor.

Silencing of the dLGN and pulvinar was performed by injecting 30 nl of 5.5 mM muscimol-BODIPY at a speed of 300 nl min^–1^, using a bevelled glass pipette (tip diameter, ~20–40 μm) on a UMP3 microinjector with a Micro4 controller (World Precision Instruments). The injector was mounted on a micromanipulator (Luigs & Neumann) for stereotactic injection. In two of the pulvinar silencing experiments, TTX was used instead. The concentration of TTX was 60 μM, and 40 μl was injected at a speed of 40 μl min^–1^. After recording, brains were fixed in 4% PFA in PBS overnight at 4 °C for histological analysis of BODIPY the next day.

### Histology

Anaesthetized mice were perfused transcardially with 4% PFA in PBS (pH 7.4). Brains were removed and further postfixed in 4% PFA in PBS at 4 °C overnight, after which the solution was replaced with PBS. They were kept at 4 °C until they were coronally sectioned (100-μm sections) with a Vibratome. Sections were mounted in Vectashield mounting medium containing DAPI (Vector Laboratories, H1500) and imaged with a camera (Olympus, DP72) attached to an MVX10 stereoscope (Olympus).

### Analyses

#### Detection of saccades

For head-fixed mice, saccades were detected post hoc from the eye tracking data, using a custom-written algorithm in MATLAB. The algorithm searched for any event in which the angular position of the eye changed by more than 0.75° along the horizontal axis in one video frame (5 ms). We discarded all events where the eye position did not move in the same direction for at least three successive frames (15 ms) and in which the peak amplitude of the eye movement was below 3°. Furthermore, to eliminate the influence of preceding saccades on V1 responses, we only analysed saccades that occurred in isolation, that is, that were preceded by a period of at least 500 ms during which the eye did not move.

In freely moving animals, a custom algorithm searched for events in which the eye position changed by more than 5.5° in any direction in one video frame (11 ms). This equates to 500° per second, exceeding the speed of most head movements in mice^58^ and thus ensuring that the detected eye movements were not image-stabilizing movements (that is, vestibulo-ocular reflexes). The beginning of the saccade was defined as the first frame in which eye movement speed exceeded 200° per second. The saccades were required to be at least two frames long (22 ms), and the vectors of the eye movement between successive frames in a saccade event were required to be within 45° of each other.

#### Unit isolation

Single units from extracellular recordings were isolated using KiloSort^59^ and visualized using Phy for further manual merging and splitting. The quality of the isolated units was assessed using refractory period violations and stability of amplitude. The depth for each unit was assigned according to the electrode site at which its amplitude was the largest. For V1 recordings, units with trough-to-peak times longer than 0.5 ms were categorized as regular-spiking neurons. Units with shorter trough-to-peak times were categorized as fast-spiking neurons. Multi-units were defined as the collection of all units that remained after excluding noise using Phy. In the main text, we refer to isolated single units as neurons.

We used the spontaneous FR to register the recording depth across experiments. We approximated the border between layer 4 and layer 5 at ~125 μm above the channel with maximum spontaneous FR. Channels within 200 μm below this border were assigned to layer 5, and channels within 150 μm above the border were assigned to layer 4.

#### Inclusion criteria

Only animals with at least 15 saccades in each direction were analysed. For this study, we focused on the saccade-related activity of V1 neurons. Nonetheless, we found single units in our recordings whose activity correlated with stationary eye position (putative ‘eye position units’), in both control and TTX-blinded animals. Because there is a correlation between the direction of saccades and the position of the eye along the horizontal plane before the saccade (that is, the more temporal the position of the eye before the saccade, the more likely the upcoming saccade will be nasal), some of these units were capable of discriminating the direction of future saccades, regardless of whether they responded to saccade onset. While these units represent a minority of the population, they would introduce a confounder in the current study because, rather than discriminating saccade direction, they code for eye position. Thus, for analyses of single units in head-fixed mice, we excluded putative eye position units, that is, units whose baseline activity (measured 500 ms before the onset of saccades) was significantly different between the two directions of the upcoming saccades (nasal and temporal). These typically accounted for 1–5% of all units in each recording. In freely moving experiments, all units were considered.

#### Response to saccades and pseudo-saccades

Saccades in freely moving animals were categorized into eight evenly spaced directions. To determine whether a unit was responsive to saccades, we proceeded as follows: we performed a Kruskal–Wallis test using the response and baseline activity of the unit in each of the eight directions (total of 16 categories). Response was defined as the number of spikes within 100 ms of the onset of saccades, while baseline activity was defined as the number of spikes in a 100-ms window from −300 ms to −200 ms with respect to saccade onset. If the unit passed this test (critical value, 0.05), we proceeded to perform multiple comparisons among the 16 categories using Tukey’s honestly significant difference procedure. A unit was considered responsive if the average response to any of the eight directions was 50% above or below the average baseline activity for the corresponding direction and met at least one of the following two criteria: (1) presence of a significant difference between baseline and response for at least one direction and (2) presence of a significant difference between the responses to any two of the eight directions.

In head-fixed experiments, units were considered responsive to saccades if they met either one of the following two criteria: (1) if the number of spikes elicited within 100 ms of saccade onset was significantly different from baseline for either the nasal or temporal direction (baseline was calculated as the number of spikes within a 100-ms window from −300 ms to −200 ms with respect to saccade onset) or (2) if the number of spikes elicited within 100 ms of saccade onset was significantly different between the nasal and temporal directions. Statistical significance was determined by rank-sum test. To account for multiple comparisons, we controlled the false discovery rate to 10% using *q* values.

All reported responses in the main text are average FRs within the 100-ms window following saccade onset unless otherwise noted.

#### Direction selectivity and discriminability

The NT discriminability of each single unit was calculated as the area under the receiver operating characteristic curve (AROC), linearly rescaled to range from −1 to 1 (Gini coefficient), that is, 2 × AROC – 1. NT discriminability was calculated on the basis of two directions, nasal and temporal. The order was fixed, such that negative values indicate a preference for temporal saccades and positive values indicate a preference for nasal saccades; that is, the sign of NT discriminability corresponds to the preferred direction. We calculated the discriminability using two series of values: (1) the number of spikes induced by each nasal saccade and (2) the number of spikes induced by each temporal saccade. The number of induced spikes was calculated as the total number of spikes within the first 100 ms of saccade or pseudo-saccade onset without baseline subtraction. In freely moving animals, the preferred direction was defined as the direction with the maximum average FR within the first 100 ms of saccade onset. The discriminability index was calculated as the absolute value of the Gini coefficient between the preferred direction and the non-preferred direction (direction opposite to the preferred direction). The statistical significance of discriminability was calculated using a rank-sum test comparing the two series of values used to calculate discriminability itself, and the false discovery rate was controlled to be below 10% using *q* values. The direction selectivity index (Extended Data Fig. 1) was defined as (*R*_pref_ – *R*_non-pref_)/(*R*_pref_ + *R*_non-pref_), where *R*_pref_ and *R*_non-pref_ are the number of spikes within the first 100 ms of saccade onset in the preferred and non-preferred directions, respectively.

#### Average PETH with baseline normalization

When generating average PETHs with baseline normalization, neurons with a baseline below 0.5 Hz were excluded to avoid substantial biases resulting from extremely low FR. The baseline of each neuron to saccades or pseudo-saccades was calculated using its mean activity 500 ms to 200 ms before onset. For other visual stimuli, mean activity between −200 and 0 ms relative to saccade onset was used. Note that this process was applied for visualization purposes only, and all statistics such as direction discriminability, the direction selectivity index and the differences in evoked FRs were calculated using all relevant neurons. The statistical significance of the difference between PETHs for the preferred and non-preferred direction was calculated for each 20-ms bin. This was calculated by signed-rank test, and statistical significance was determined by setting the false discovery rate to be below 10% through the Benjamini–Hochberg procedure.

#### Modelling of saccade response on a vertical grating with visual and non-visual inputs

Saccade responses on a vertical grating (the number of evoked spikes within 100 ms of saccade onset) were predicted from (1) pseudo-saccade response, (2) saccade response on a grey screen or (3) the sum of the two responses. All responses were baseline-subtracted values. The model is a linear regression (fivefold cross-validated) with no intercept, followed by thresholding, which ensured that the predicted FR did not fall below 0 Hz. That is, if the predicted decrease in the evoked number of spikes exceeded the baseline FR, the value was adjusted so that the sum of the prediction and the baseline was zero. The explained variance is calculated as the explained sum of squares divided by the total sum of squares.

#### Identification of pulvinar neurons with axonal projections to V1 through antidromic activation

V1 was illuminated with 1-ms-long pulses (100 trials) from a 465-nm blue LED to induce antidromic spikes (see above). Success of antidromic activation was defined by two criteria: (1) greater than 20% probability of observing at least one spike within 5 ms of the onset of LED illumination across trials and (2) less than 0.5 ms jitter (that is, the s.d. of the latency distribution of the first spikes occurring within the 5-ms window following LED onset was less than 0.5 ms).

#### Classification of saccade direction in head-fixed mice

We classified the direction of saccades and pseudo-saccades using quadratic discriminant analysis (QDA) on the response of each single unit. The spiking activity of each unit was counted in 20-ms bins, and the activity at 60 ms after onset for each event was taken as the response. The discriminant analysis was preceded by principal-component analysis (PCA) for dimensionality reduction. Only single units with average FR above 0.5 Hz were used. For each event of saccades or pseudo-saccades, the classifier assigned either nasal or temporal direction.

Training data consisted of the response to selected pseudo-saccades. This set of pseudo-saccades was selected such that the amplitudes and number of events for the nasal and temporal directions were matched. This ensured that the classifier depended on the NT discriminability of each unit, rather than on the difference in pseudo-saccade amplitude or frequency. The training dataset was first standardized and subjected to PCA. We limited the number of principal components to 20% of the total number of saccades in the training dataset to avoid overfitting. We then trained QDA for classification. The resulting models for PCA and QDA were applied to the test dataset, which comprised responses to either real saccades or pseudo-saccades that were excluded from the training dataset (10-fold cross-validation).

To pool single units recorded from multiple animals, we closely matched the direction and amplitude of the pseudo-saccades for each animal (see ‘Visual stimulation’). From this dataset, we further generated a random subset in which the amplitudes for the nasal and temporal pseudo-saccades were closely matched. Ten such datasets were generated to be used as training datasets. For the test dataset, saccade data from different animals were pooled on the basis of the direction and amplitude of saccades, again such that the directions and amplitudes were closely matched between animals.

To calculate classifier performance as a function of the number of single units used for classification, a random subset of units (5, 10, 15, 20, 30, 40, 50, 100, 175 or 250 units) was chosen from the pooled data without replacement, before being subjected to training and testing. Random selection of units was repeated 50 times, for every randomly generated training dataset (see above), resulting in 500 results that were averaged to calculate decoder performance.

To rank the contribution of each unit to the classifier model, we calculated the permutation feature importance. In brief, we permuted the data from one unit at a time in the pseudo-saccade training dataset during 10-fold cross-validation, to break the relationship between unit activity and pseudo-saccade direction. We then calculated the increase in prediction error resulting from the permutation procedure. To calculate the total contribution from single units with the highest feature importance, we permuted the data from the corresponding units at the same time.

### Reporting summary

Further information on research design is available in the Nature Research Reporting Summary linked to this article.

## Online content

Any methods, additional references, Nature Research reporting summaries, source data, extended data, supplementary information, acknowledgements, peer review information; details of author contributions and competing interests; and statements of data and code availability are available at 10.1038/s41586-022-05196-w.

## Supplementary information

Reporting Summary

## Data Availability

Processed data are available at 10.7272/Q6513WG4. Raw data (eye tracking movies, electrophysiology data and others) will be made available upon reasonable request.

## Code availability

Custom codes written in LabVIEW (2013 SP1 f2) and MATLAB (R2013b/R2022a) used to generate and analyze data are available at https://github.com/Satoru-k-miura/Miura-and-Scanziani-Nature-2022. 
