# Publisher Correction: Distinguishing externally from saccade-induced motion in visual cortex

**DOI:** 10.1038/s41586-022-05428-z

**Published:** 2022-10-14

**Authors:** Satoru K. Miura, Massimo Scanziani

**Affiliations:** 1 Center for Neural Circuits and Behavior, Neurobiology Section and Department of Neuroscience, University of California, San Diego, La Jolla, CA USA; 2 Department of Physiology, University of California, San Francisco, San Francisco, CA USA; 3 Howard Hughes Medical Institute, University of California, San Francisco, San Francisco, CA USA

**Keywords:** Neuroscience, Physiology

Correction to: *Nature* 10.1038/s41586-022-05196-w Published online 14 September 2022

In the version of this article initially published, a spurious image of a pipette was directed toward the eye image in the lower section of Fig. 5a. The pipette was placed in error and has been removed from the HTML and PDF versions of the article.

