# Supplementary material for: Distinguishing externally from saccade-induced motion in visual cortex
Source: Nature. 2022 Sep 14;610(7930):135–42. doi: 10.1038/s41586-022-05196-w (PMC9534749; doi:10.1038/s41586-022-05196-w)
Supplement: Supplementary file 1 — Reporting Summary [file 41586_2022_5196_MOESM1_ESM.pdf]

nature research

Corresponding author(s): Satoru K Miura, Massimo Scanziani

Last updated by author(s): Jul 17, 2022

Reporting Summary

Nature Research wishes to improve the reproducibility of the work that we publish. This form provides structure for consistency and transparency in reporting. For further information on Nature Research policies, see our Editorial Policies and the Editorial Policy Checklist.

Statistics

For all statistical analyses, confirm that the following items are present in the figure legend, table legend, main text, or Methods section.

n/a	Confirmed	
☐	☒	The exact sample size (*n*) for each experimental group/condition, given as a discrete number and unit of measurement
☐	☒	A statement on whether measurements were taken from distinct samples or whether the same sample was measured repeatedly
☐	☒	The statistical test(s) used AND whether they are one- or two-sided<br>*Only common tests should be described solely by name; describe more complex techniques in the Methods section.*
☒	☐	A description of all covariates tested
☐	☒	A description of any assumptions or corrections, such as tests of normality and adjustment for multiple comparisons
☐	☒	A full description of the statistical parameters including central tendency (e.g. means) or other basic estimates (e.g. regression coefficient) AND variation (e.g. standard deviation) or associated estimates of uncertainty (e.g. confidence intervals)
☐	☒	For null hypothesis testing, the test statistic (e.g. F, t, r) with confidence intervals, effect sizes, degrees of freedom and P value noted<br>*Give P values as exact values whenever suitable.*
☒	☐	For Bayesian analysis, information on the choice of priors and Markov chain Monte Carlo settings
☒	☐	For hierarchical and complex designs, identification of the appropriate level for tests and full reporting of outcomes
☐	☒	Estimates of effect sizes (e.g. Cohen's *d*, Pearson's *r*), indicating how they were calculated

Our web collection on statistics for biologists contains articles on many of the points above.

Software and code

Policy information about availability of computer code

Data collection	Intan system and software (version 1.5, Intan Technologies); Psychophysics Toolbox (PTB-3, Open source); custom code written in Matlab (2013b, 2018a, MathWorks); custom code written in LabVIEW (2013 SP1 f2 64bit, National Instruments). Custom codes will be made available on a public repository for download.
Data analysis	Data were analyzed with custom code written in Matlab (2013b, 2018a, 2022a; MathWorks) and will be made available for download from a public repository prior to final publication. Electrophysiology recordings were preprocessed using KiloSort and Phy.

For manuscripts utilizing custom algorithms or software that are central to the research but not yet described in published literature, software must be made available to editors and reviewers. We strongly encourage code deposition in a community repository (e.g. GitHub). See the Nature Research guidelines for submitting code & software for further information.

Data

Policy information about availability of data

All manuscripts must include a data availability statement. This statement should provide the following information, where applicable:

- Accession codes, unique identifiers, or web links for publicly available datasets
- A list of figures that have associated raw data
- A description of any restrictions on data availability

Datasets supporting the findings of this paper will be made available on a public repository for download

Field-specific reporting

Please select the one below that is the best fit for your research. If you are not sure, read the appropriate sections before making your selection.

☒ Life sciences ☐ Behavioural & social sciences ☐ Ecological, evolutionary & environmental sciences

For a reference copy of the document with all sections, see nature.com/documents/nr-reporting-summary-flat.pdf

Life sciences study design

All studies must disclose on these points even when the disclosure is negative.

Sample size	No statistical methods were used to pre-determine sample sizes. Sample sizes reported in this paper were chosen to be similar to those used in previous publications in the field.
Data exclusions	Only animals with at least 15 saccadic events in each direction (i.e., nasal and temporal) were analyzed to ensure sufficient statistical power. A minor fraction of neurons whose firing rates depended significantly on the eye position were excluded from further analyses, as this study primarily concerns the impact of saccade-triggered inputs on the ability of the neurons to discriminate the saccade direction. This is because the starting position of the eye confines the possible direction of the future eye movement, and thus eye position information introduces a confounder. The criteria are fully described in the Methods under the subsection "Inclusion criteria" in "Analyses".
Replication	Multiple independent samples were collected for each experiment, and all attempts to replicate the data were successful. The number of samples for each experiment are indicated in the manuscript.
Randomization	Only one genotype of mice (C57BL/6J) was used throughout the study, and males and females were randomly assigned.
Blinding	Data collection and analyses were not performed blind to the experimental conditions.

Reporting for specific materials, systems and methods

We require information from authors about some types of materials, experimental systems and methods used in many studies. Here, indicate whether each material, system or method listed is relevant to your study. If you are not sure if a list item applies to your research, read the appropriate section before selecting a response.

Materials & experimental systems

n/a	Involved in the study	
☒	☐	Antibodies
☒	☐	Eukaryotic cell lines
☒	☐	Palaeontology and archaeology
☐	☒	Animals and other organisms
☒	☐	Human research participants
☒	☐	Clinical data
☒	☐	Dual use research of concern

Methods

n/a	Involved in the study	
☒	☐	ChIP-seq
☒	☐	Flow cytometry
☒	☐	MRI-based neuroimaging

Animals and other organisms

Policy information about studies involving animals; ARRIVE guidelines recommended for reporting animal research

Laboratory animals	C56BL/6J mice, both males and females, between ages 3 to 6 months were used. They were kept in a 12:12 hour dark:light reverse light cycle at room temperature (~20-22C) and ~40-60% humidity.
Wild animals	This study did not involve wild animals.
Field-collected samples	This study did not involve field-collected samples.
Ethics oversight	All experiments were conducted in accordance with the regulations of the Institutional Animal Care and Use Committee of the University of California, San Diego and of the University of California, San Francisco.

Note that full information on the approval of the study protocol must also be provided in the manuscript.
